# Vinegar and Flesh

**Published:** 1875-03

**Authors:** 


VINEGAR AND FLESH.—A little vinegar used with the class of food which seems to need it, is not injurious; used in excess, it does great harm. Experiments on artificial digestion show that if the quantity of acid be diminished, digestion is retarded; if increased beyond a certain point, digestion stops. This accounts for the theory that vinegar keeps down flesh; for, by drinking it, fat people may become thin. It will take off the flesh, and it will just as surely take health and life with it. To take acid enough to reduce the flesh is simply to destroy the power of digestion. A few years since, a young lady in easy circumstances enjoyed good health; she was very plump, had a good appetite, and a complexion blooming with roses and lilies. She began to look upon her plumpness with suspicion, for her mother was very fat, and she was afraid of becoming like her. Accordingly, she consulted a woman, who advised her to drink a glass of vinegar daily. The young lady followed her advice, and her plumpness diminished. She was delighted with the success of the experiment, and continued it for more than a month. She began to have a cough; but it was dry at its commencement, and was considered as a slight cold which would go off. Meantime, from dry it became moist; a slow fever came on, and a difficulty of breathing; her body became lean and wasted away; night sweats, swelling of the feet and of the legs succeeded, and a diarrhœa terminated her life. Therefore, young ladies, be boldly fat! Never pine for graceful slimness and romantic pallor; but if nature intends you to be ruddy and rotund, accept it with a laughing grace, which will captivate more hearts than all the paleness of a circulating library. But remember, that small eaters, who take only two meals in twenty-four hours and nothing between, do not get fat, unless they are very indolent and walk but little. It is easy enough to keep off excessive flesh by a reasonable system of living, but it is not as easy to get rid of it after it has become the habit.

---

YOUNG PEOPLE left to the government of themselves commence to read the sickening trash printed in story papers without a particle of evil intent. After a while they get to be absorbed in them, and then commences the vitiating of the mind and the formation of a regular passion for the closest perusal of every scrap that comes under the observation of the eye. What follows such a course of reading? Ask the youth who attends low theatres and immoral places of amusement. Ask the ballet-girls whose supreme indifference to modesty renders their appearences so hateful to every well regulated mind, and so enticing for the moment to the depraved tastes of those who devour the illustrated literature of the cities. Ask the easy-minded fathers and mothers of families who are left alone to weep over the frailties of their children.

---

What is time? The shadow on the dial, the striking of the clock, the running of the sand, day and night, summer and winter, months, years, centuries: these are but arbitrary and outward signs, the measure of time, not time itself. Time is the life of soul. If not this, then tell me what is time?

